# The effect of an HIV-1 viral protease inhibitor on staurosporine-induced apoptosis in immortalized mesangial cells

**DOI:** 10.1590/S1516-31802002000300005

**Published:** 2002-05-02

**Authors:** Adrian Pierce Serone, Simone Mafalda Rodrigues Camargo, Nestor Schor

**Keywords:** Immortalized mesangial cell, Progressive glomerular sclerosis, Apoptosis, Célula mesangial imortalizada, Glomeruloesclerose progressiva, Apoptose

## Abstract

**CONTEXT:**

Progressive glomerular sclerosis is a condition characterized by the accumulation of glomerular extracellular matrix and a decrease in the number of glomerular cells. The mechanisms involved in the progressive loss of glomerular cells are not well understood but may involve the process of apoptosis. The principal mediators for the apoptotic pathway are a class of protease enzymes called caspases. It is not known how other therapeutic protease inhibitors affect the caspase cascade and therefore whether they would be effective in preventing excessive apoptosis in the late stages of progressive glomerular sclerosis.

**OBJECTIVE:**

To evaluate whether an inhibitor of the HIV-1 viral protease Ac-Leu-Val-phenylalanine (PI) could inhibit apoptosis in immortalized mesangial cells.

**DESIGN:**

Experimental.

**SETTING:**

Nephrology Division, Universidade Federal de São Paulo/Escola Paulista de Medicina.

**PARTICIPANTS:**

Immortalized mesangial cells.

**PROCEDURES:**

Cell culture.

**MAIN MEASUREMENTS:**

Viability and rate of apoptosis.

**RESULTS:**

Immortalized mesangial cells were treated with staurosporine (at concentrations of 10-100 nM for 8-28 hours) to induce apoptosis. Staurosporine at 10 nM for 8 hours had no effect on viability, but did cause a significant increase in the rate of apoptosis (p = 0.0411, n = 6). Increasing the incubation time elicited a greater increase in the rate of apoptosis (p = 0.0001, n = 6), although there was also a significant decrease in viability (p=0.0002). Increasing the concentration of staurosporine to 100 nM resulted in a marked increase in apoptosis (p <0.0001) but resulted in unacceptable viability (<40%, p <0.0001, n = 6).

**CONCLUSIONS:**

Incubation of immortalized mesangial cells with PI (900 nM) alone for 2-24 hours had no effect on cell viability or the rate of apoptosis when compared with vehicle (methanol) controls. Co-incubation of the cells with staurosporine (10 nM) and PI for 24 hours had no significant effect on the rate of apoptosis. Therefore, in immortalized mesangial cells, staurosporine-induced apoptosis was not significantly affected by the HIV-1 viral protease inhibitor Ac-Leu-Val-phenylalanine.

## RESUMO

**CONTEXTO:**

A glomeruloesclerose progressiva (GEP) é uma situação caracterizada pela acumulação de matriz extracelular glomerular e pela diminuição no número de células no glomérulo. Os mecanismos envolvidos na perda progressiva do número glomerular da célula não são bem compreendidos, mas podem envolver o processo da apoptose. Os principais mediadores na evolução da apoptose são uma classe de enzimas de protease chamadas caspases. Não se sabe como outros inibidores de proteases com potencial uso terapêuticos efetuam a cascata dos caspases e, conseqüentemente, se podem ser eficazes em impedir a apoptose nos estágios anteriores da glomeruloesclerose progressiva.

**OBJETIVO:**

Avaliar se um inibidor da protease HIV-1 viral (Ac-Leu-Val-phenylalanine; o PI) poderia prevenir a apoptose em células mesangiais glomerulares imortalizadas.

**TIPO DE ESTUDO:**

Estudo experimental

**LOCAL:**

Disciplina de Nefrologia da Universi-dade Federal de São Paulo/Escola Paulista de Medicina, São Paulo, Brasil.

**PARTICIPANTES:**

Células Mesangiais Imortalizadas.

**PROCEDIMENTOS:**

Cultura de Célula.

**VARIÁVEIS ESTUDADAS:**

Viabilidade e taxa de apoptose.

**RESULTADOS:**

As células mesangiais imortalizadas foram tratadas com a estaurosporina (10-100 nM por 8 a 28h) para induzir a apoptose. A estaurosporina (10 nM; 8 h) não teve nenhum efeito viável, mas causou um significativo aumento na taxa de apoptose (p = 0,0411, n = 6). O aumento do tempo de incubação proporcionou um grande aumento da taxa de apoptose (p = 0,0001, n = 6), entretanto, há também uma significativa diminuição da viabilidade (p = 0,0002). O aumento da concentração de estaurosporina 100 nM resultou em aumento da apoptose (p <0,0001), porém com níveis inaceitáveis de viabilidade (< 40%, p <0,0001, n = 6).

**CONCLUSÕES:**

A incubação de células mesangiais imortalizadas com PI (900 nM) isolado por 2-24 h não teve efeito na viabili-dade celular nem na taxa de apoptose comparada aos meios de controle (metanol). Coincubação das células com estaurosporina (10 nM) e PI por 24 horas não adicionou efeito significativo na taxa de apoptose. Conseqüentemente, nas células mesangiais imortalizadas a apoptose induzida por estaurosporina não foi afetada significativamente pelo inibidor viral da protease HIV-1 Ac-Leu-Valphenylalanine.

## INTRODUCTION

Progressive glomerular sclerosis (PGS) is a condition that results from a variety of glomerular injuries and is characterized by the prominent pathological features of accumulation of glomerular extracellular matrix (ECM) and decrease in the number of glomerular cells. Whilst the mechanisms responsible for the accumulation of glomerular extracellular matrix have been well studied and are thought to involve growth factors such as transforming growth factor-b,^1,2^ the mechanisms involved in the progressive loss of glomerular cells are less well understood.^3,4^ One possible mechanism for the observed decrease in the number of glomerular cells may involve the process of apoptosis.^2,5,6^

Apoptosis (also referred to as programmed cell death) is a genetically programmed series of events that, through activation of a final common biochemical pathway, ultimately leads to the dismantling of the cell into easily digestible membrane-intact packages (with the notable absence of the inflammation that is common in necrosis).^5,7^ Morphologically, apoptosis is defined by a shrinkage in cell volume and the dissociation of the cell from its neighbors, maintenance of the integrity of intracellular organelles and the plasma membrane, and most distinctively, the condensation of nuclear chromatin. In the final stages of apoptosis, the condensed nucleus fragments to form membrane-bound vesicles (apoptotic bodies), which are then phagocytosed by neighboring cells.^7^

The principle mediators in the apoptotic pathway are a class of protease enzymes called caspases.^8-10^ These enzymes derive their name from the fact that they all have cysteine in their active site and cleave their target proteins at specific aspartic acids.^8^

Whilst there are no caspase inhibitors that are currently available for therapeutic use, there are currently two classes of protease inhibitors that have been utilized in the treatment of some pathologies; the inhibitors of the HIV-1 viral protease and angiotensin-converting enzyme inhibitors. It is not known how these drugs may affect the caspase cascade and therefore whether they would be effective in preventing excessive apoptosis and thus glomerular cell loss in the late stages of progressive glomerular sclerosis.

Our aim was therefore to evaluate whether an inhibitor of the HIV-1 viral protease (AcLeu-Val-phenylalanine) could inhibit apoptosis in immortalized mesangial cells.

## METHODS

Immortalized mesangial cells (CRL-1927), were obtained from the American Type of Culture Collection (ATCC) and were cultured from passages 43 to 50 in Dulbecco's Modified Eagle Medium (DMEM), supplemented with fetal bovine serum (FBS; 5%), NaHCO_3_ (2 g/l), HEPES (2.6 g/l), penicillin (10,000 IU/l) and streptomycin (50 mg/ml). Cells were cultured in either polystyrene bottles or 24-well culture plates at 37°C in a humidified gas mixture (95% air and 5% CO_2_).

Cell viability was assessed by the exclusion of the fluorescent dyes acridine orange (AO) and ethidium bromide (EB).^11^ Apoptosis was determined morphologically using a fluorochrome [bis-benzimide Hoe 33342 (2’-(ethoxyphenyl)-5-(4-methyl-1-piperazinyl)-2,5’-bis-benzimidazole, HCl)] (HOE 33342, 50 μg/ml). Cells presenting with condensed nuclei and/or apoptotic bodies were counted as positive for apoptosis.

Previously, protein kinase inhibition had been shown to lead to apoptosis in many different cell types.^12^ The mechanism by which this occurs is believed to be via arresting cells at the G1 checkpoint.^12^ We used the broad spectrum protein kinase inhibitor staurosporine (shown to inhibit CaM kinase, myosin light-chain kinase, protein kinase A, protein kinase C and protein kinase G) to induce apoptosis in immortalized mesangial cells. Cells were treated with 10-100 nM of staurosporine for 8-28 hours and then their viability and apoptosis were assessed.

## RESULTS

**Figure 1:**
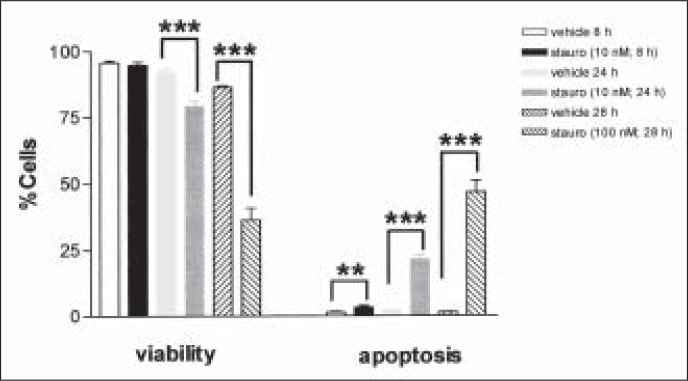
Effect of the broad-spectrum protein kinase inhibitor staurosporine (stauro, n = 6 per group) or vehicle (DMSO, n = 6 per group) on viability and apoptosis in immortalized mesangial cells in culture. **, p <0.01; ***, p <0.001, 1-way ANOVA with Bonferroni post-hoc test for multiple comparisons.

Treatment of immortalized mesangial cells with staurosporine at 10 nM for 8 hours caused no significant difference in viability when compared with vehicle (DMSO) controls (Figure 1), but did elicit a small increase in the rate of apoptosis (p = 0.0411, n = 6; Figure 1). When the incubation time was increased to 24 hours, staurosporine at 10 nM caused a significant decrease in viability (p = 0.0002) and increase in apoptosis (p = 0.0001, n = 6; Figure 1) when compared with vehicle. When exposed to a tenfold greater concentration of staurosporine (100 nM) for 28 hours, the viability of immortalized mesangial cells was markedly reduced to below 40% (p <0.0001, n = 6; Figure 1), whilst the rate of apoptosis rose to almost 50% (p <0.0001).

## DISCUSSION

A significant effect was observed from the vehicle alone, when evaluating cell viability (p <0.0001). From the above data, it was determined that treatment of immortalized mesangial cells with staurosporine at 10 nM for 24 hours appeared to achieve the maximum increase in the rate of apoptosis whilst still retaining a cell viability level of greater than 75%. Therefore, this treatment regimen was to be utilized as the principle method of inducing apoptosis for testing the effect of protease inhibition in the subsequent experiments.^13,14^

**Figure 2:**
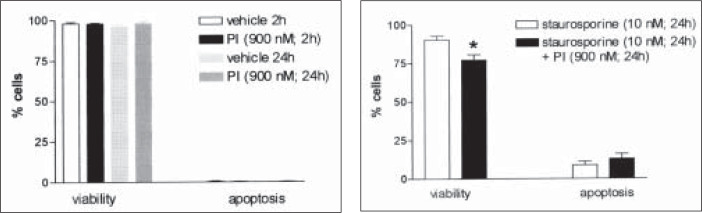
Effect of the HIV-1 viral protease inhibitor Ac-Leu-Val-phenylalanine (PI) or vehicle (methanol) on viability and apoptosis in the absence and presence of the broad-spectrum protein kinase inhibitor staurosporine (n = 4 per group). *, p <0.05; 1-way ANOVA with Bonferroni post-hoc test for multiple comparisons.

The effect of the HIV-1 protease inhibitor Ac-Leu-Val-phenylalanine (PI; Calbiochem, CA, USA) on the rate of staurosporine-induced apoptosis was then examined. Cells were cultivated in 24-well plates as described above. After reaching a confluence of around 80-90%, the cells were treated for 24 hours with one of the following: i) vehicle (methanol, 20 μl); ii) staurosporine (10 nM); iii) protease inhibitor (900 nM – this concentration is the *p*CI_50_ for this drug); iv) staurosporine (10 nM) plus protease inhibitor (900 nM). Incubation of immortalized mesangial cells with PI alone for 2 hours had no acute effect on cell viability or the rate of apoptosis, when compared with vehicle controls (Figure 2). Increasing the time of incubation to 24 hours (to coincide with the incubation time required for staurosporine-induced apoptosis) had no significant effect on either the viability or rate of apoptosis (Figure 2). Incubation of the cells with 10 nM staurosporine, which was the concentration shown to induce a significant amount of apoptosis in preliminary experiments, again caused a significant rise in the rate of apoptosis observed, with a slight decrease in the viability. However, in these experiments the effect of staurosporine alone on inducing apoptosis in immortalized mesangial cells was significantly less than in the preliminary initial experiments (Figures 1 and 2). The reason for this decreased effectiveness of staurosporine is not understood. The only difference between the two protocols is the fact that in these latter experiments, staurosporine was dissolved in a methanol vehicle, whilst previously the staurosporine had been prepared as a solution with viability comparable to that of the vehicle. Therefore, whilst it is unlikely, the difference in vehicle may be causing a difference in the potency of this drug. Co-incubation of the cells with staurosporine and PI for 24 hours had no significant effect on the rate of apoptosis, although cell viability was significantly decreased in comparison with staurosporine alone (p = 0.0222, n = 4; Figure 2).

## CONCLUSION

It could therefore be seen that, in immortalized mesangial cells, staurosporine-induced apoptosis was not significantly affected by the HIV-1 viral protease inhibitor Ac-Leu-Valphenylalanine. This suggests that this protease inhibitor would be ineffective in preventing the late stages of progressive glomerular sclerosis.
